# Computational modeling of Takotsubo cardiomyopathy: effect of spatially varying β-adrenergic stimulation in the rat left ventricle

**DOI:** 10.1152/ajpheart.00443.2014

**Published:** 2014-09-19

**Authors:** Sander Land, Steven A. Niederer, William E. Louch, Åsmund T. Røe, Jan Magnus Aronsen, Daniel J. Stuckey, Markus B. Sikkel, Matthew H. Tranter, Alexander R. Lyon, Sian E. Harding, Nicolas P. Smith

**Affiliations:** ^1^Department of Biomedical Engineering, King's College London, London, United Kingdom;; ^2^Institute for Experimental Medical Research, Oslo University Hospital Ullevål, Oslo, Norway;; ^3^KG Jebsen Cardiac Research Center and Center for Heart Failure Research, University of Oslo, Oslo, Norway;; ^4^National Heart and Lung Institute, Imperial College London, London, United Kingdom;; ^5^National Insitute of Health Research Cardiovascular Biomedical Research Unit, Royal Brompton Hospital, London, United Kingdom; and; ^6^Faculty of Engineering, University of Auckland, Auckland, New Zealand

**Keywords:** Takotsubo cardiomyopathy, cardiac modeling, catecholamine overload

## Abstract

In Takotsubo cardiomyopathy, the left ventricle shows apical ballooning combined with basal hypercontractility. Both clinical observations in humans and recent experimental work on isolated rat ventricular myocytes suggest the dominant mechanisms of this syndrome are related to acute catecholamine overload. However, relating observed differences in single cells to the capacity of such alterations to result in the extreme changes in ventricular shape seen in Takotsubo syndrome is difficult. By using a computational model of the rat left ventricle, we investigate which mechanisms can give rise to the typical shape of the ventricle observed in this syndrome. Three potential dominant mechanisms related to effects of β-adrenergic stimulation were considered: apical-basal variation of calcium transients due to differences in L-type and sarco(endo)plasmic reticulum Ca^2+^-ATPase activation, apical-basal variation of calcium sensitivity due to differences in troponin I phosphorylation, and apical-basal variation in maximal active tension due to, e.g., the negative inotropic effects of p38 MAPK. Furthermore, we investigated the interaction of these spatial variations in the presence of a failing Frank-Starling mechanism. We conclude that a large portion of the apex needs to be affected by severe changes in calcium regulation or contractile function to result in apical ballooning, and smooth linear variation from apex to base is unlikely to result in the typical ventricular shape observed in this syndrome. A failing Frank-Starling mechanism significantly increases apical ballooning at end systole and may be an important additional factor underpinning Takotsubo syndrome.

takotsubo cardiomyopathy is a severe form of heart failure characterized by acute apical dysfunction, in which the shape of the left ventricle resembles the Japanese fisherman's octopus pot (tako-tsubo) during systole. This condition is typically associated with sudden emotional or physical stress (2) and has been recognized in survivors of major natural disasters including earthquakes, hurricanes, and tornadoes.

While the majority of patients recover without intervention in a period of days to weeks, there are currently limited treatment options available and serious complications may occur during the acute phase, with associated morbidity and a 1–3% mortality rate (6, 19). Misdiagnosis and incorrect treatment are frequent, as the syndrome mimics symptoms of acute myocardial infarction. Current data on prevalence show ∼2% of suspected acute coronary syndromes are eventually diagnosed as Takotsubo cardiomyopathy (6), with ∼7,000 cases per year in the United States (5). Prevalence is higher in postmenopausal women, where recent data show 5.9% of acute coronary syndrome admissions are eventually diagnosed as Takotsubo (29).

Furthermore, the disease is especially common in postmenopausal women (∼90% of reported patients) with the majority being over 50 yr old (6). Sudden cardiac death related to similar high stress situations has an ∼80% male bias (18), raising the possibility that events that cause Takotsubo cardiomyopathy in postmenopausal women may be fatal in men. Such differences make this syndrome potentially important for understanding gender differences in heart failure as well as sudden cardiac death in general.

This context motivates an improved mechanistic understanding of this condition. However, this detailed understanding of the disease is currently lacking. Multivessel coronary vasospasm has been suggested as a possible cause (6), including in the original report by Sato et al. (24). However, further research showed that <1.5% of Takotsubo patients are affected by this (2). A common factor in most current hypotheses is the high level of the catecholamines epinephrine and norepinephrine (also known as adrenaline and noradrenaline), which have been measured in patients (2, 16, 20).

Results from a recent animal study have also pointed to epinephrine-induced stunning of the myocardium as a potential mechanism (16, 20). Infusion of high epinephrine into anaesthetized rats reproduced the basal hypercontractility and apical hypokinesia that underlie the appearance of apical ballooning in the clinical syndrome. Exposing isolated cardiac cells with human β_2_-receptors to high concentrations of epinephrine cause a decrease in force generated by these cells (8). Similarly, challenging apical cells from normal rats with high epinephrine suppressed the increases in contraction observed with normal levels of epinephrine (20). However, it was not clear whether strong negative effects were required in the apex to produce ballooning or whether the high contractility in the base, coupled with an unstimulated contraction in the apex, can produce the same phenomenon.

Although a decrease in tension at high levels of epinephrine has been shown experimentally, the biophysical mechanisms underpinning this phenomenon remain poorly understood. For β_2_- or β_2_-adrenergic receptor (AR) stimulation in the normal physiological range, G_s_ protein activates the adenylyl cyclase → cyclic AMP (cAMP) → protein kinase A (PKA) pathway. PKA phosphorylation stimulates L-type calcium channels leading to more calcium influx and increased contraction, while phosphorylation of phospholamban removes the inhibitory effect on the sarco(endo)plasmic reticulum Ca^2+^-ATPase (SERCA) pump and phosphorylation of troponin I (TnI) decreases calcium sensitivity, both of which aid relaxation. The resulting calcium and force transients have a higher peak but also relax more rapidly. By contrast, at supraphysiological levels of epinephrine, contraction of cardiac myocytes starts to decrease instead, as the β_2_-AR signaling pathway switches from the G_s_ pathway to the G_i_ pathway (8). This pathway blocks cAMP production, thereby reversing some of the effects of G_s_ stimulation, and also targets downstream pathways, which have been shown to promote cell survival. We have previously hypothesized that there is an additional negative inotropic effect as a result of these other pathways (20). Thus the apical stunning appears to be a side-effect of preventing cardiac cells from dying of the toxic effects of extreme levels of epinephrine. One such signaling molecule that has been implicated in the decreased tension is p38 mitogen-activated protein kinase (p38 MAPK), which is known to be involved in many cellular processes including the proliferation, growth, and death of cells across many species (14). p38 MAPK has also been shown to significantly reduce contractility of cardiac cells without affecting the calcium transients or calcium sensitivity (30).

Given that differences in β_2_-AR stimulation are the likely cause of Takotsubo cardiomyopathy, an explanation of the observed apical ballooning requires both a mechanism for the decrease in tension, as well as quantitative spatial differences in these changes in tension generation. Detailed studies of such apical-basal differences in catecholamines are rare, but measurements indicate that samples from the basal myocardium show a higher norepinephrine content, which is likely due to a higher density of sympathetic nerve endings, as well as a higher proportion of β_1_-ARs (21). By contrast, the apex has been shown to have a higher proportion of β_2_-ARs, compensating for the lower levels of locally released norepinephrine by increased sensitivity (1). Overall there appears to be a balance between catecholamine release and receptor density, which is likely to cause the effects of β-adrenergic stimulation to be homogeneous under physiological conditions, with the apical myocardium being more sensitive to the effects of circulating epinephrine released by the adrenal glands. However, these subtle differences between the apex and base are also a potential substrate for Takotsubo cardiomyopathy. Thus the mechanisms of Takotsubo cardiomyopathy are likely to involve the different effects that the β-adrenergic stimulants epinephrine and norepinephrine have on G-protein pathways (8) (changes in calcium transients, calcium sensitivity, and decreased tension), in combination with differences in apical-basal density of sympathetic nerve endings and receptor densities. However, many aspects of the complex signaling networks remain poorly understood, and the necessary quantitative gradients in force generation required to produce the observed apical ballooning have not yet been studied.

The goal of the research presented in this article is to investigate what the biophysical requirements are for generating the typical “takotsubo” morphology, both in terms of potential dominant change in protein regulation and the spatial gradient required to produce an apical ballooning phenotype. For this purpose we have developed a quantitative measure of apical ballooning and a series of biophysically based computational models of the left ventricle. Using these models we investigate three hypotheses. We test in a model whether apical ballooning can be generated if the dominant mechanism is apical-basal differences in the calcium transients induced by catecholamine overload. Secondly, we test apical-basal differences in calcium sensitivity as a dominant mechanism, and thirdly, we test apical-basal differences in maximal active tension due to, e.g., cross-bridge inhibition from p38 MAPK as a dominant mechanism. The computational model used in our study is of a rat ventricle, for maximal compatibility with available experimental data for wild-type animals and the recent development of the rat as an animal model for Takotsubo cardiomyopathy (8, 20).

## MATERIALS AND METHODS

For our computational model, we use our standard framework based on a finite element model of solid mechanics for the ventricle, coupled to a system of ordinary differential equations that predict the amount of force produced by cardiac myocytes. The finite element code and biophysical contraction model are based on our previous work in computational methods and mouse modeling (11, 13). Our computational model of the rat left ventricle was based on experimental measurements of calcium, tension, left-ventricular pressure-volume (PV), and cardiac geometry. The next two sections describe both the experimental methods for obtaining data and the computational methods used for integrating these data in a model of the rat heart.

### 

#### Experimental methods.

Experimental protocols for PV and MRI measurements were approved by an institutional ethics committee and carried out in accordance with the *Guide for the Care and Use of Laboratory Animals* published by the U.S. National Institutes of Health (NIH) under Assurance No. A5634-01. All animal surgical procedures and perioperative management conformed to the UK Animals (Scientific Procedures) Act 1986. Cardiac cine-MRI was performed as previously described (28). Briefly, rats were anesthetized with 1.5% isoflurane in O_2_ and placed supine in a 4.7-T DirectDrive MRI system (Agilent Technologies) with a 72-mm quadrature-driven birdcage RF coil (Agilent). Cardiac and respiratory gated cine-MRI was performed in the two-chamber long axis and true short-axis orientations and covered the whole left ventricle (8–10 × 1.5 mm slices, TE/TR 1.6/5 ms; 15° flip angle; field of view 51.2 × 51.2 mm; matrix size 192 × 192; voxel size 267 × 267 × 1,500 μm; 30–35 frames per cardiac cycle, 3 signal averages). The left ventricle in these MRI measurements was manually segmented and used to generate a computational geometry in the form of a finite element mesh.

PV loop studies were performed under general anesthesia (1.5% isoflurane) while the animal was intubated. A 1.9-F Scisense PV catheter attached to the ADVantange acquisition system (Scisense, ON, Canada) was introduced into the left ventricular apex via a subcostal incision. Data were recorded using Labchart 6 software (AD Instruments, Oxford, UK) and analyzed offline using PVAN 3.6 software (Millar Instruments). Steady-state measurements were attained under conditions of spontaneous cardiac contraction with no intervention applied to the animal and comprise ejection fraction and left ventricular end diastolic pressure. These PV measurements were used for setting end-diastolic pressure and target systolic pressure and Windkessel parameters for the model described in the next section.

**Fig. 1.**
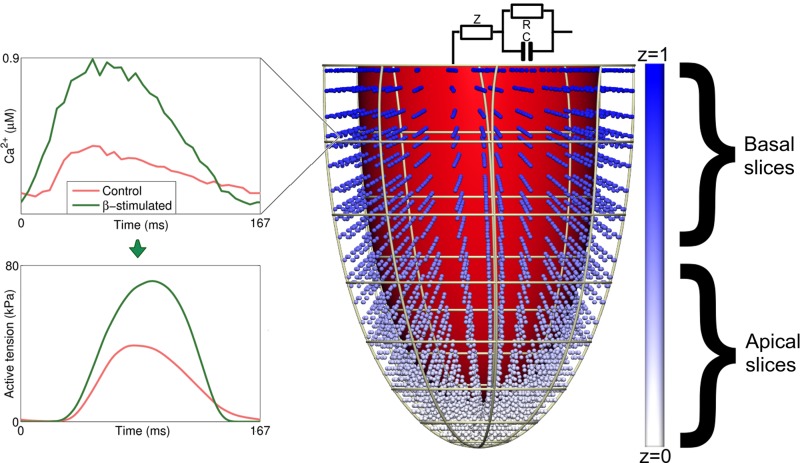
Schematic for computational methods. Shown here is the computational mesh used, the linear gradient in the variable *z* that defines the apex-base position for defining spatial gradients (blue to white spheres as well as the color bar on the *right*). Indicated on the *right* are the slices used for defining our maximal apical radius/minimal basal radius (A:B) metric for apical ballooning in *Eq. 1*. Plots on the *left* show the representative 6-Hz experimental rat myocyte calcium transients used to drive contraction in the computation.

Experimental protocols for calcium and force measurements were approved by a local ethics committee and were performed in accordance with the Norwegian Animal Welfare Act, which conforms to NIH guidelines (NIH Publication No 85-23, revised 1996). Cardiomyocytes were enzymatically isolated (15), loaded with fluo-4 AM (Invitrogen Molecular Probes, Eugene, OR), and plated in a perfusion chamber on an inverted microscope. Cells were superfused with HEPES Tyrode's solution containing the following (in mM): 140 NaCl, 1.8 CaCl_2_, 0.5 MgCl_2_, 5.0 HEPES, 5.5 glucose, 0.4 NaH_2_PO_4_, and 5.4 KCl (37°C, pH 7.4) and field stimulated at 6 Hz. Ca^2+^ transient measurements were recorded by whole cell photometry (Photon Technology International Monmouth Junction) in the absence and presence of 100 nM isoproterenol. Fluorescence measurements were calibrated to Ca^2+^ concentration using the method of Cheng et al. (4). Representative calcium transients (Fig. 1) were used to drive the computational model.

Muscle strips were dissected out from the left ventricle and mounted between metal clips in an organ bath. The bath was initially filled with a relaxing solution containing the following (in mM): 118.3 NaCl, 3 KCl, 0.2 CaCl_2_, 4.0 MgSO_4_, 2.4 KH_2_PO_4_, 24.9 NaHCO_3_, 10.0 glucose, and 2.2 mannitol. A pH of 7.4 was maintained by bubbling with 95% O_2_-5% CO_2_. The experimental solution was identical to the relaxing solution, except with 1.2 mM MgSO_4_ and 1.8 mM CaCl_2_ (23). During 1-Hz field stimulation (37°C), strips were stretched until maximal force development was observed and then allowed to equilibrate until stable mechanical performance was obtained (∼1 h). Force was then recorded during 0.5-, 1-, 2-, 4-, and 6-Hz stimulation. The 6-Hz measurements were used to parametrize the computational model described in the next section.

#### Computational methods.

Cellular calcium transients measured at 6-Hz pacing with and without isoproterenol were used to drive the model, as in our previous work on characterizing the β-adrenergic response (12). Specifically, we prescribed synchronous activation and did not include inherent spatial variation in calcium transients beyond the apical-basal gradients described in the next section. The contraction model was reparametrized based on experimental force measurements. Specifically, based on differences in myosin isoforms and calcium transients between mice and rats, we adjusted the rate of cross-bridge cycling *k*_*xb*_ = 0.02 to fit measurements on time-to-peak (experimental range 39 ± 6 ms) and 50% relaxation time (31 ± 5 ms) for isometric twitch force. Calcium sensitivity Ca_T50_ was also adjusted as described in the next sections. In our model, the parameter Ca_T50_ is the half-activation value of troponin C for calcium, denoted [Ca^2+^]_T50_^ref^ in previous work describing the contraction model used (13). This parameter directly influences the half-activation value for tension generation EC_50_, such that relative changes in Ca_T50_ are equal to relative changes in EC_50_. The whole organ model uses material parameters equal to our previous mouse models (13), as changes in inflation from PV were consistent with these parameters. Hemodynamic parameters for the three-element Windkessel model were based on a review by Westerhof and Elzinga (31), with the aortic compliance adjusted to *C* = 0.0144 ml/mmHg to better match observed maximum pressure (≈15 kPa), end diastolic volume (100–150 μl), and ejection fraction (≈75%) seen in experimental PV transients.

For the simulation of Takotsubo cardiomyopathy, we created specific models that each test a dominant mechanism being activated in a spatially dependent manner.

**Fig. 2.**
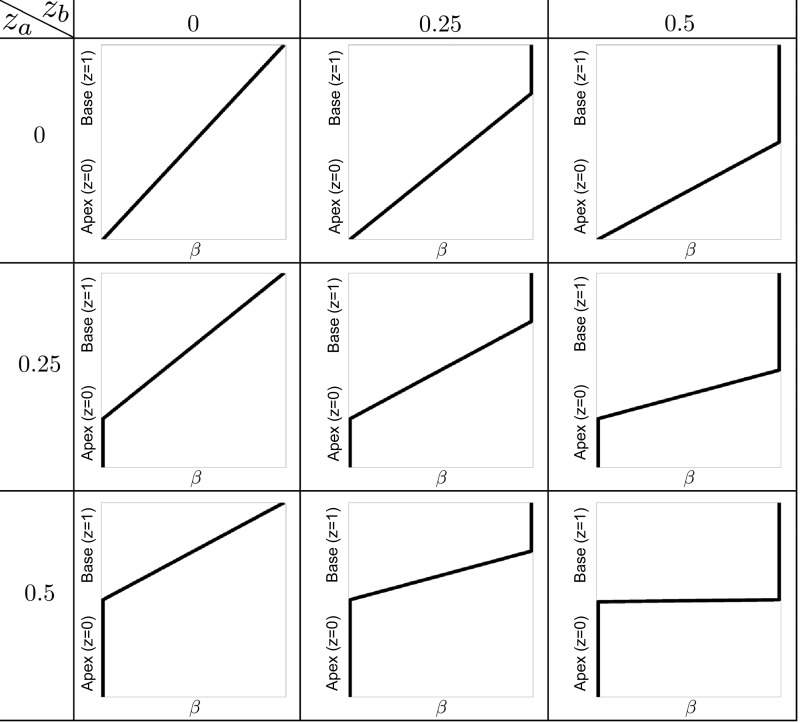
Spatial gradients. The different spatial gradients used throughout this article for use in parameter variation studies. These range from a gradual linear change in parameters to 2 regions with a sharp transition. The parameter *z*_*a*_ represents the fraction of the ventricle at the apex that is minimally activated and *z*_*b*_ the fraction of the ventricle at the base that is maximally activated. These are referred to as the size of the apical region and the size of the basal region. We define a parameter β, which varies from β = 0 at z ≤ *z*_*a*_ to β = 1 at *z* ≥ 1 − *z*_*b*_ and is used to drive the variation in β-adrenergic stimulation, with a linear variation between these regions. This allows us to test both gradual changes from apex to base, e.g., *z*_*a*_ = *z*_*b*_ = 0, as well as sharp differences as given by, e.g., *z*_*a*_ = 0.5, *z*_*b*_ = 0.5.

For all three models, we map a variable z ranging from 0 at the apex to 1 at the base, and two parameters *z*_*a*_ and *z*_*b*_. The variable *z* is shown in Fig. 1, with the different spatial variation patterns defined using *z*, *z*_*a*_, *z*_*b*_ shown in Fig. 2. Parameter variation in both model parameters and spatial variations within individual simulations collectively provide an extensive exploration of the quantitative effects of spatial variation in a specific pathway. The three variations on the contraction model described in the next sections are available in CellML format at http://models.cellml.org/workspace/1b9.

#### Changes in calcium transients as dominant mechanism.

Changes in L-type calcium channel regulation and SERCA regulation result in changes in the calcium transients, which include both higher peak and lower diastolic calcium levels. If spatial differences in these effects are dominant in producing apical ballooning, such upregulation needs to appear mainly in the base of the ventricle, while high catecholamine levels at the apex reverse the effects of the upregulation as described in the introduction. To produce apical stunning, a decreased calcium sensitivity is also required. Our first computational model tests this hypothesis by prescribing cellular calcium transients that vary from apex to base due to differences in adrenergic stimulation. The properties of this gradient were varied in this model study in combination with the calcium sensitivity of the myofilaments. The calcium transient varies from normal at β = 0 to maximal β-adrenergically stimulation at β = 1. That is, for the apical region (*z* < *z*_*a*_) we use the normal calcium transient, for the basal region (*z* > 1 − *z*_*b*_) the β-adrenergically stimulated transient, and in between we use a weighted combination of both calcium transients determined by linear interpolation. Apical stunning in this model is driven by a combination of a normal calcium transient with lower calcium sensitivity (i.e., higher Ca_T50_), where the latter is set to be homogeneous throughout the ventricle. This represents a heart in which due to β-toxicity the L-type calcium channel and SERCA pump revert back to their normal state, whereas TnI phosphorylation is maintained consistent with a high β-adrenergic level. We vary parameters *z*_*a*_, *z*_*b*_ as shown in Fig. 2, as well as eight different calcium sensitivity settings between Ca_T50_ = 0.6 − 1.5 μM for a total of 72 simulations.

#### Changes in calcium sensitivity as dominant mechanism.

The change in calcium sensitivity through TnI phosphorylation is another important mechanism in the response to catecholamines. A decrease in calcium sensitivity decreases the amount of force generated at identical calcium levels. Large changes in calcium sensitivity could result in apical stunning. However, to produce a hypercontractile base, an increased calcium transient also needs to be present in the basal myocardium. Our second model tests this hypothesis by prescribing spatial gradients in calcium sensitivity. We also apply the β-adrenergically stimulated calcium transient throughout the myocardium to simulate a general high level of β-adrenergic stimulation. Again, we use the same threshold system, with highest calcium sensitivity Ca_T50_ = 1 μM (and therefore lower contractility) at β = 0 and lowest calcium sensitivity at β = 1. We vary parameters *z*_*a*_, *z*_*b*_ as shown in Fig. 2, as well as 10 different apical calcium sensitivity settings ranging from Ca_T50_ = 1.0 − 4.0 μM for a total of 90 simulations.

#### Changes in maximum active tension as dominant mechanism.

As described in the Introduction, another proposed pathway for apical ballooning is through tension inhibition produced by p38 MAPK. These experiments typically show a change in tension generation without a change in calcium sensitivity Thus a change in the reference tension *T*_ref_, which is proportional to the maximum active tension a cell generates at saturating calcium, functions as a good phenomenological model for negatively inotropic responses that do not affect the calcium transient, time to peak, or relaxation time. This includes the type of response seen experimentally as a result of p38 MAPK. We use the same threshold system with lower maximum tension near the apex at β = 0 and normal maximum tension at β = 1. We vary parameters *z*_1_, *z*_2_ as shown in Fig. 2, as well as apical *T*_ref_ = 30, 60, and 90 kPa (compared with 120 kPa normally, i.e., 75, 50, and 25% cross-bridge inhibition, respectively), and eight different settings for calcium sensitivity between Ca_T50_ = 0.6 − 1.3 for a total of 216 simulations.

#### Defining a measure for apical ballooning.

The previous sections described the parameters and spatial gradients explored in this study, resulting in nearly 400 full-cycle cardiac simulations. The resulting data set is too large to fully present here in the same way that is usual in work using electromechanical models. To select results to visualize and present in this article, and objectively measure the amount of apical ballooning, we now propose a quantitative metric of apical ballooning. This metric will be used in the rest of the article to select results to present, and compare the effect of spatial variations and parameters on the shape of the ventricle. Specifically, to characterize the difference in apical and basal contraction at end systole, we consider the difference in radius for a given cross section of the ventricle at the apex and the base, specifically the ratio of radius at the apex and base defined as:

(1) $$\text{A}:\text{B=}\frac{\underset{\text{apical}\;\;\text{slices}}{\text{max}}\;r_{e}}{\underset{\text{basal}\;\;\text{slices}\;}{\text{min}}\;r_{e}}$$

where *r*_e_ is the mean distance from the endocardial surface to the center of the cavity for a given cross section at end systole. Figure 1 shows the slices used for the “apical” and “basal” slices in our computational mesh. Applying this metric to a typical example of human MRI and echo data in the literature (see Fig. 1, *A* and *E* in Ref. 2) results in A:B ≈ 1.35 − 2.0.

## RESULTS

### 

#### Relation between the ejection fraction and apical ballooning.

**Fig. 3.**
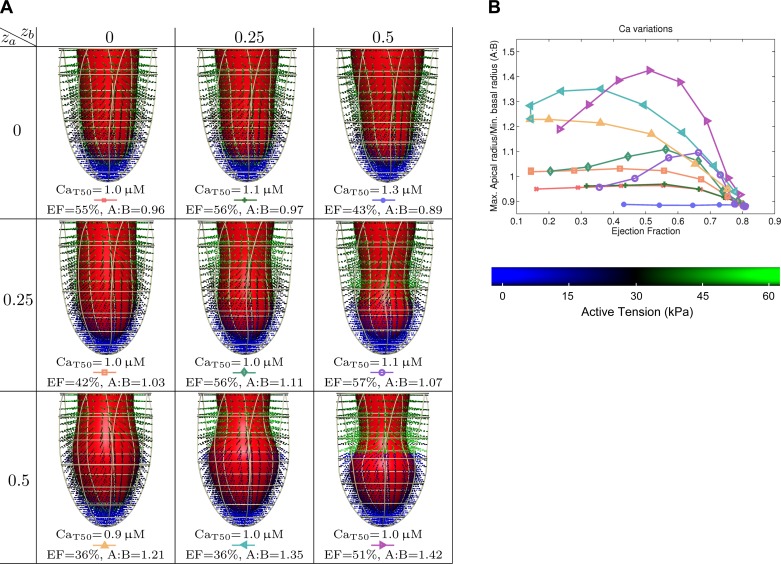
Results for spatial variations in calcium transients. *A*: ejection fraction (EF) vs. maximal apical radius/minimal basal radius (A:B). *B*: details of selected results, with end-systolic geometry, calcium sensitivity (Ca_T50_), EF, and our metric of apical ballooning (A:B). Active tension generation is visualized in the Gaussian integration points of the computational geometry. These 9 results are ordered by apical/basal regions, as in Fig. 2.

**Fig. 4.**
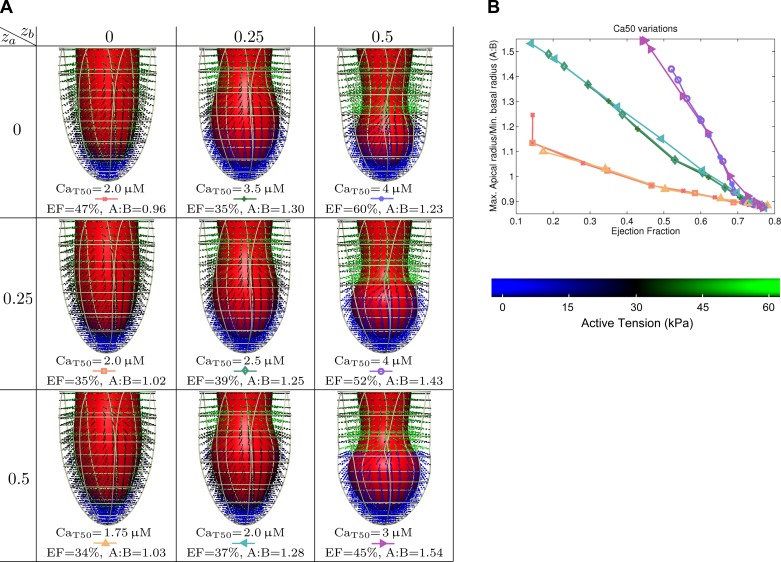
Results for spatial variations in calcium sensitivity. *A*: EF vs. maximal apical radius/minimal basal radius (A:B). *B*: details of selected results, with end-systolic geometry, calcium sensitivity (Ca_T50_), EF, and our metric of apical ballooning (A:B).

**Fig. 5.**
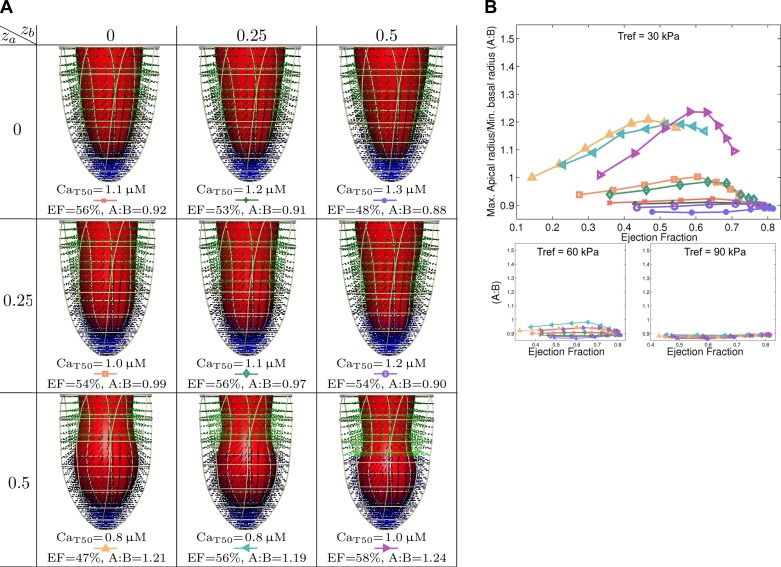
Results for spatial variations in maximum active tension. *A*: EF vs. maximal apical radius/minimal basal radius (A:B). *B*: details of selected results.

Based on the A:B metric defined above, we start with a high-level analysis of the relation between model parameters and apical ballooning. Shown in the graphs in Figs. 3–5 are the relation between apical ballooning and left-ventricular ejection fraction. These curves generally have a peak A:B measure at an ejection fraction between 30 and 60%. At lower ejection fraction, neither apex nor base ejects, and at higher ejection fraction, both regions tend to eject almost equally well. We can also see that there is significant variation in apical ballooning depending on the spatial gradient, with sharper gradients having a tendency for a higher values of the A:B metric. Based on this result, as well as the observation of moderate to severe ejection fraction reduction in Takotsubo cardiomyopathy (2), we will focus on the result with maximal A:B and ejection fraction between 30 and 60% in the next sections. Secondly, for variations in the maximum active tension *T*_ref_, we only analyze the *T*_ref_ = 30 kPa case representing 75% apical cross-bridge inhibition, as it is the only case that shows significant apical ballooning.

#### Model results.

Results for spatial variations in calcium transients selected for an ejection fraction between 30 and 60% are shown in Fig. 3. These show that a combination of a high spatial gradient in adrenergic effects on the calcium transient, combined with a homogeneous decrease in calcium sensitivity, is sufficient to reproduce mild apical ballooning. Results for spatial variations in calcium sensitivity in Fig. 4 show a very typical “takotsubo” shape for sharper gradients, although very low apical calcium sensitivity is required. Results for spatial variations in maximum active tension are shown in Fig. 5, where only moderate apical ballooning was achieved.

#### Effects of an impaired Frank-Starling mechanism.

**Fig. 6.**
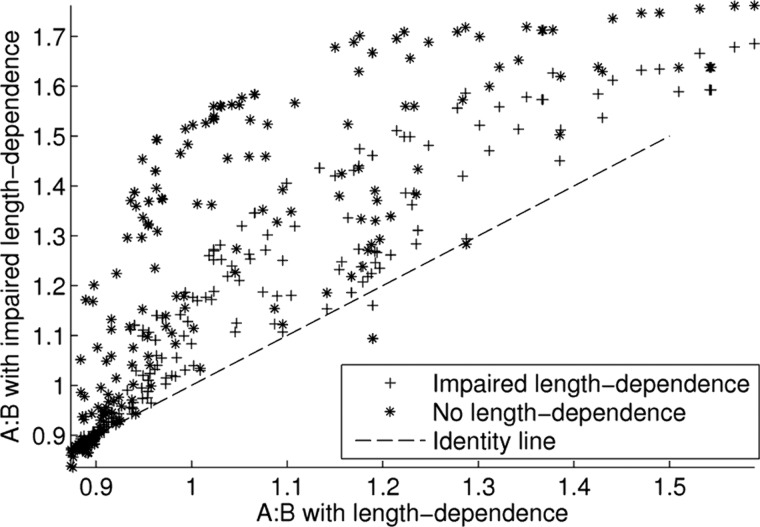
Effect of impaired length dependence of tension. Shown are the effects of halved and fully removed length dependence on apical ballooning as measured by our “A:B” index. A reduction in the length dependence of tension generation creates a significant increases in apical ballooning in many cases.

**Fig. 7.**
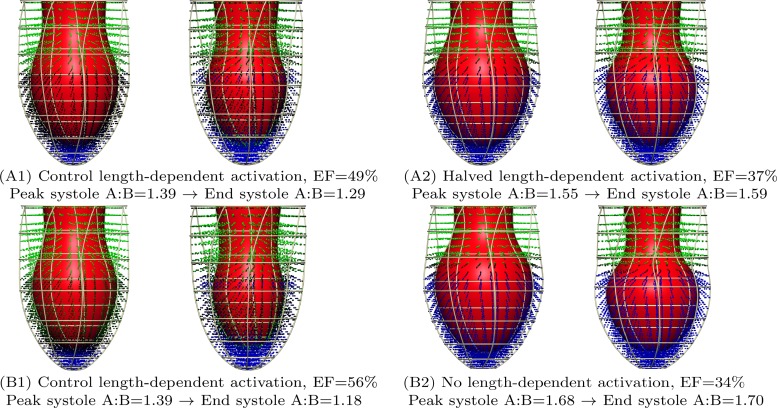
Examples of maximal increase in end-systolic apical ballooning after a decrease in length-dependent activation (LDA). Exampled were limited to those simulations with an ejection fraction of ≥30% after the change in parameter, and chosen to represent the maximal relative increase in A:B at end systole after changing the degree of LDA. Both cases represent apical-basal variations in the calcium transient. *A1* and *A2*: case with maximal change in the A:B metric when halving the LDA parameter from β_1_ = −1.5 to β_1_ = −0.75, which has parameters *z*_*a*_ = 0.5, *z*_*b*_ = 0, Ca_T50_ = 0.9 μM. *B1* and *B2*: case with maximal change in the A:B metric when setting the LDA parameter to β_1_ = 0, which has parameters *z*_*a*_ = 0.25, *z*_*b*_ = 0, Ca_T50_ = 0.8 μM.

In many cases studied in the previous sections, the amount of apical ballooning decreases significantly between mid- and end systole. We hypothesized this effect to be due to compensation produced by the length dependence of tension in cardiac muscle, the same mechanism that is responsible for the Frank-Starling response of the heart. Reports of Takotsubo cardiomyopathy feature significant apical ballooning at end systole, and changes in length dependence of tension have been suggested to be related to both heart failure and β-adrenergic stimulation (7). Driven by these observations we investigated the effect of an impaired length dependence of tension in maintaining apical ballooning at end systole. To test the effects of an impaired length dependence of tension, we changed the magnitude of the length-dependent change in calcium sensitivity from β_1_ = −1.5 to β_1_ = −0.75 and β_1_ = 0. All simulations from the previous section were run again with this change in parameter. Figure 6 shows the effect of an impaired length dependence of tension on the A:B index. Note the large number of cases that now show significant apical ballooning which previously showed normal contraction motion without apical ballooning. Figure 7 shows some selected examples where this change is maximal, including the clear differences in the decrease in A:B throughout systole.

#### Effects of increased afterload.

**Fig. 8.**
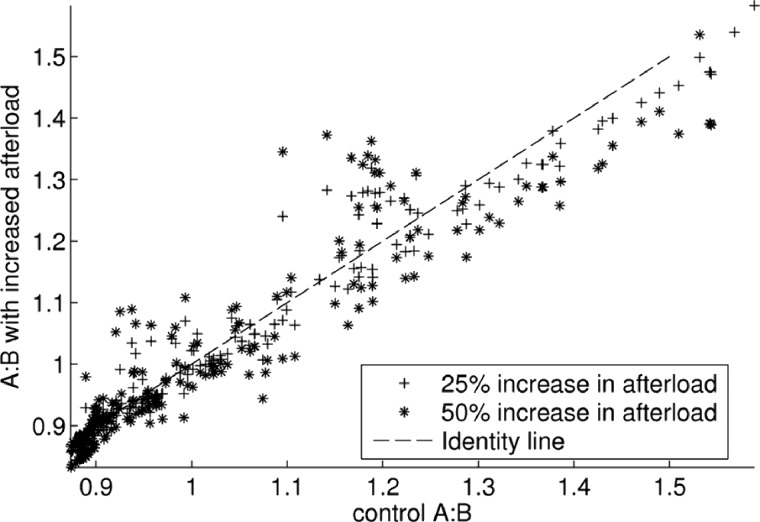
Effect of increased afterload. Figure shows the effects of 25 and 50% increased afterload as measured by our “A:B” index. Increased afterload is simulated by the pressure at which ejection starts, which represents the minimal aortic pressure and is given by *p*_*a*_ = 9 kPa control, 11.25, and 13.5 kPa in the increased cases, respectively.

Afterload plays an important role in determining ejection fraction, duration of systole, and systolic pressure. All three factors have the capacity to influence the shape of the ventricle in the face of gradients in calcium sensitivity, calcium transients, and maximal tension. Recent research in rat models of Takotsubo syndrome has suggested blood pressure may be an important factor to consider (22). To test this factor, all simulations from the previous section were run again with an increase in afterload. Figure 8 shows the effect of an impaired length dependence of tension on the A:B index, which shows that this parameter can increase A:B by around ∼0.2 in selected cases.

## DISCUSSION

Our goal in this investigation was to quantify the necessary spatial variations for generating a “Takotsubo” shape, for several dominant mechanisms of end-point protein regulation from apex to base. We investigated changes in calcium transient, calcium sensitivity, and maximum active tension, based on changes to calcium dynamics caused by β-adrenergic stimulation and recent observations of the effects of p38 MAPK after catecholamine overload. To manage the large number of simulations, we have proposed the A:B metric for apical ballooning that is simply the ratio of the maximal apical and minimal basal radius. This metric works well in practice and corresponds to an intuitive sense of apical ballooning in visualizations.

Our results show that generating significant apical ballooning at end systole requires large variations in active tension, especially in the presence of a healthy Frank-Starling mechanism. Applying an apical-basal variation in the calcium transient generated significant apical ballooning (A:B ≥ 1.3) only in two cases. Apical ballooning requires a large apical region spanning ∼50% of the ventricle where contraction is driven by the control calcium transient as well as a large (25–50%) basal region with a β-adrenergically stimulated calcium transient. Smoother transitions do not generate apical ballooning, with results showing that a gradual linear variation or smaller regions result in a maximal A:B close to 1, indicating relatively homogeneous shortening despite significant differences in apical and basal calcium transients. We have also shown that significantly reduced calcium sensitivity is required to reproduce observed apical ballooning and that, in addition to not reproducing the observed reduction in ejection fraction, an increase in basal calcium transients does not produce apical ballooning.

For our results in investigating variations in calcium sensitivity, the sharpest transition again produces the most significant apical ballooning of all cases and mechanisms. Producing this amount of ballooning requires a large decrease in calcium sensitivity (3 μM, which is 5–6× the nonstimulated level), as the hypercontractile base causes large stretch in the boundary region, which activates the apical region through the Frank-Starling mechanism. This directly compensates for changes in calcium sensitivity, and limits apical ballooning. Smoother spatial variations such as a 25–50% apical region and a 25% basal region, which are physiologically more plausible, also result in significant ballooning. However, the change in apical calcium sensitivity needs to be significantly lower than the basal calcium sensitivity, which is already lower than the nonstimulated level. The total change in calcium sensitivity is thus relatively high compared with differences shown to be caused by phosphorylation in the literature, e.g., up to about three times the nonstimulated level as measured by in vitro motility assays (3), making changes in calcium sensitivity a less plausible dominant mechanism for generating apical ballooning.

The last mechanism we tested is related to changes in the maximum active tension. This change was based on observations of lower active tension without changes in calcium sensitivity for myocytes exposed to p38 MAPK. We found that ≥75% cross-bridge inhibition in the apical 50% of the heart is needed to cause significant apical ballooning. Unlike the other two cases, apical ballooning is similar in some of the smoother variations compared with the case produced by a sharp transition halfway between apex and base. These result can be explained by considering that decreases in maximum tension are not as well compensated for by the Frank-Starling mechanisms. Even though calcium sensitivity is increased through length dependence, tension development is still limited by the decreased maximum tension. By contrast, for changes in calcium sensitivity, length-dependent effects can fully compensate to result in the same effective apical tension. There are currently limited data on the amount of tension reduction caused by p38 MAPK. Vahebi et al. (30) showed a 25% reduction in maximum tension in transgenic mice but test only a single concentration caused by inserting an upstream activator. Liao et al. (14) showed a 240% increase in contractility when p38 MAPK is suppressed, as measured by shortening of muscle in a twitch activation, but this is difficult to relate to maximum tension.

Based on these observations, we can make the following conclusions about the likely mechanisms underpinning the Takotsubo phenotype. A sharp transition halfway through the ventricle is the most straight-forward model perturbation for generating apical ballooning. However, given the proposed hypotheses for the apical-basal variations, i.e., different receptor densities and catecholamines release, this gradient is also physiologically less plausible. Many of the spatial gradients tested, including a smooth linear gradient, do not result in any observable apical ballooning as mechanisms related to the length dependence of tension tend to compensate. As a smooth gradient is clearly not sufficient to produce apical ballooning by any tested mechanism, an experimental study measuring the spatial gradients in the heart would benefit from four to five measurements along the long axis of the ventricle, instead of assuming a smooth gradient.

We performed a wide range of simulations of impaired length dependence of tension, as such changes have been suggested to be related to both heart failure and β-adrenergic stimulation (7). Our results showed that an impaired length dependence of tension significantly increases apical ballooning. Thus preexisting conditions or acute changes to the Frank-Starling mechanism may make patients more susceptible to Takotsubo cardiomyopathy. Given that there is currently a limited understanding of the mechanisms underlying the length dependence of tension, it is difficult to identify the pathway responsible for such changes with certainty. Differences in titin isoforms have been implicated in changes in length-dependent activation (17), but these changes are too slow to form part of an acute response, although they may contribute to apical ballooning in the case of preexisting conditions. By contrast, changes to phosphorylation of myofilament proteins including TnI have been shown to decrease length-dependent changes in calcium sensitivity (9) and are thus more likely to form part of an acute decrease in length dependence leading to apical ballooning. Finally, we tested the effects of an increased afterload. Although an increase in blood pressure can moderately increase apical ballooning in selected cases, no significant change is observed in most simulations.

Replicating the amount of apical ballooning observed in the human clinical cases in a consistent animal model is challenging, and current results typically show less apical ballooning than clinical cases in the literature (20). However, our results do reveal a potential explanation of why investigations have found difficulty applying the rat as an animal model in Takotsubo cardiomyopathy. For human patients, a healthy ejection fraction is ∼60%, whereas in rats this metric tends towards 75–80% (27). In this study we have shown that high contractility and ejection fraction can result in lower apical ballooning. This may be an important factor in the difficulty of inducing apical ballooning in rats in vivo.

A limitation of our study is that we have studied each potential mechanism independently, while the actual effects of catecholamine overload are likely to be multifactorial and may include mechanisms not studied in our models such as changes in peripheral vascular response or other hemodynamic parameters. Takotsubo syndrome is also associated with ECG changes including ST elevation, T wave inversion, and QTc prolongation (10). Although the current mechanisms causing the ECG changes are unclear, these changes have been hypothesized to be due to changes to K_ATP_ (25) and other potassium channels not represented in the current model, rather than changes in deformation that have only limited impact on the ECG (26). Given the limited data about spatial heterogeneity in acute catecholamine overload, this was necessary to limit the number of simulations to be performed and presented. As more data become available on the effects, our framework can be readily applied to analyzing hypotheses based on multifactorial mechanisms. This lack of detailed data on quantitative measurements of in vivo differences in apical-basal adrenergic stimulation has also limited validation of the computational model, as many of its predictions cannot yet be directly compared with experimental data. However, the ability of the model to reproduce both cellular data and PV measurements from healthy animals and the addition of data from isoproterenol-stimulated cells limit the uncertainty in the ability of the models to represent changes in calcium dynamics.

In conclusion, our model has shown the quantitative gradients in tension generation required to produce apical ballooning consistent with Takotsubo cardiomyopathy. This has resulted in clearer requirements for specific hypotheses related to catecholamine overload and led to a framework that can be used to interpret future experimental data in this area.

## GRANTS

This work was supported by Biotechnology and Biological Sciences Research Council (BB/J017272/1), Virtual Physiological Rat Project (NIH 1 P50 GM094503-01), and Engineering and Physical Sciences Research Council (EP/F043929/1 and EP/G007527/2). A. R. Lyon is supported by a British Heart Foundation Intermediate Research Fellowship FS/11/67/28954 and National Institute for Health Research (NIHR) Cardiovascular Biomedical Research Unit, Royal Brompton Hospital. M. H. Tranter is supported by National Heart and Lung Institute Foundation Studentship. M. B. Sikkel is supported by the Wellcome Trust (WT092852). We acknowledge financial support from the Department of Health via the NIHR comprehensive Biomedical Research Centre Award to Guy's & St Thomas' National Health Services (NHS) Foundation Trust in partnership with King's College London and King's College Hospital NHS Foundation Trust and the NIHR Cardiovascular Biomedical Research Unit at the Royal Brompton Hospital.

## DISCLOSURES

No conflicts of interest, financial or otherwise, are declared by the author(s).

## AUTHOR CONTRIBUTIONS

Author contributions: S.L., S.A.N., S.E.H., and N.P.S. conception and design of research; S.L. analyzed data; S.L., S.A.N., and N.P.S. interpreted results of experiments; S.L. prepared figures; S.L. drafted manuscript; S.L., S.A.N., D.J.S., M.B.S., A.R.L., S.E.H., and N.P.S. edited and revised manuscript; S.L., S.A.N., W.E.L., A.T.R., J.M.A., D.J.S., M.B.S., M.H.T., A.R.L., S.E.H., and N.P.S. approved final version of manuscript; W.E.L., A.T.R., J.M.A., D.J.S., M.B.S., M.H.T., and A.R.L. performed experiments.
